# A Contribution to the Study of the Continued Fevers of the South

**Published:** 1891-02

**Authors:** Llewellin P. Barber

**Affiliations:** Tracy City, Tenn.


### Southern Medical Record.

A MONHLY JOURNAL OF MEDICINE AND SURGERY.

VOL. XXI. ATLANTA, GA., FEBRUARY, 1891. NO. 2.

### Original Articles.

### A CONTRIBUTION TO THE STUDY OF THE CONTINUED FEVERS OF THE SOUTH.

BY LLEWELLIN P. BARBER, M. D., TRACY CITY, TENN.

Read before the Tri-State Medical Association, Chattanooga, Tennessee, Oct. 14th, 1890.

The continued Fever of the South—its nosology and etiology—forms a subject now justly attracting much attention, a subject upon which much is yet to be learned, and over which the medical world is considerably at variance. Only close and accurate study of the disease by competent observers, in many and differing localities, and thoughtful comparison of these observations, with free discussion will advance our knowledge of its nature, and throw light on the vexed question of its cause.

From the first days of my practice, this disease, so common to all parts of Tennessee and its adjoining States, has proved of great interest to me. Encouraged by the recent vigorous inquiry and research in this direction, I began some fifteen months since, to keep record of all fevers occurring in my private practice, and the comparison of these cases—irrespective of their different designations—has helped me toward a decision as to the nosology of continued fever. The paper which I propose to read to-day is mainly a report of a number of these cases. No plan of selection has been followed other than that the cases have occurred in one immediate neighborhood —a circle drawn around the reported district would have a diameter of not more than one fourth mile. I have also narrated them in the order of proximity and time of seizure, and the picture thus given of continued fever is exactly as it is seen by me in Tracy City. First, however, a word as to the local and sanitary conditions of the town. Tracy City is a mining town on the Cumberland Plateau, 2200 feet above sea level. Its soil is a light, porous sand, its rock a conglomerate sandstone. Some portions of the town are usually clean, but some parts are very filthy—refuse and slops being thrown about the the houses forming cess-pools, often in close proximity to the well. The water supply is mainly from wells, though some springs and a few cisterns are in use.

Now to proceed to the narration of cases, which will be given with all brevity possible, without sacrificing essential detail:

CASE I. Male, aged 18 years. Had complained for a few days of a tired feeling and slight headache. At 7 p. m., July 21, 1889, when first examined, he had considerable headache and increased sense of fatigue; temperature 102, pulse 90, tongue moist and but little coated, with dirty red line through the middle, skin dry, abdomen natural, bowels had moved from purgative taken the preceding day.

July 22, 10 a. m., temperature 101, pulse 90, tongue as day before, bowels moving naturally, skin a little moist.

July 23, 10:30 a. m., temperature 101, pulse 98, tongue moist and coated, skin moist; no delirium; 5 p. m., temperature 101 1-2, pulse 105, tongue a little dry, skin dry, no movement of bowels.

For the next five days, the patient's condition remained about the same, with a slight increase of fever; bowels moved every second or third day.

July 28, 10 a. m., temperature 102 1-2; at 5 P. M., 103, tongue not very dry, and red line less marked than earlier in the case, bowels moved once, some nausea.

July 29, temperature at 4 p. m. 103; no action from bowels.

July 30, 10 a. m., temperature 101 3-4, pulse 101, tongue and skin moist; 4:30 p. m., temperature 102 1-2, pulse 105, tongue clearing, bowels moved once.

From this date the fever lessened each day, and August 4th the temperature was normal. At no time during the progress of this case was there abdominal tenderness or tympanites, and nausea only two days when the fever was at its height.

CASE II. Male, aged 9 years. First called to see him July 22, 1889. Had complained for three or four days of headache and fatigue. At 2 p. m. temperature 102, pulse 106, tongue heavily coated with a white, furry coat, abdomen not at all tender, no tympanites, bowels moving regularly.

July 23, 2 p. m., temperature 102, pulse 108, other symptoms as day before.

July 24, 10 a. m., temperature 102, pulse 105.

July 25, 3 p. m., temperature 103, pulse 117, tongue moist, no movement of bowels.

July 26, 2 p. m., temperature 103 1-2, pulse 118.

July 27, 2. p. m., temperature 103, pulse 116, tongue coated but moist.

July 28, temperature 102 3-4, pulse 116, tongue moist, abdomen not at all tender, no movement of bowels since the 26th, no tympanites.

July 29, 3 p. m., temperature 103 1-4, pulse 116, bowels moved from oil given the day before.

July 30, temperature 103 1-4, pulse 118, tongue heavily coated. With slight variation this condition continued until August 5. On that day at 3 p. m., temperature 102 1-4, pulse 110, tongue beginning to clear. The fever lasted four days longer, and the case went on to a slow convalescence. There was no delirium at any time.

In a neighboring house to case II., I was called on the same day—July 22, to Case III., male, aged 12 years. Had complained but one day. Temperature at 3 p. m., 101, pulse 100, bowels moving a little free, abdomen slightly tender on pressure, no tympanites, tongue very little coated, but with red streak through the middle.

July 23 ,11 a. m., temperature 102, pulse 104, no movement from bowels, tongue moist and slightly coated.

July 24, 10 a. m., temperature 102, pulse 102, tongue moist.

From this date, the case ran a similar course to case II, except that the temperature was not quite as high, and there was a little tympanites and tenderness of the abdomen throughout the disease. No delirium.

CASE IV., male, aged 20 years, and of same family as case II. First visit August 5, 1889; temperature 100, pulse 85, tongue slightly coated, but large and flabby, showing indentations of teeth; no tympanites, no tenderness of bowels, considerable pain in left knee-joint but no swelling; bowels moving every day, headache.

Aug. 6, condition the same, except an increase of one-half degree in temperature; severe headache.

Aug. 7, knee-joint very painful, and slightly swollen, temperature 100 1-2, pulse 98, bowels had not moved in two days, probably due to opiate.

Aug. 8, about as previous day.

Aug. 9. Temperature 102, pulse 105, and of fair volume; tongue moist, knee-joint less painful.

Aug. 10–13 inclusive, condition about the same, with a slight rise in fever.

Aug. 14, I was hurriedly summoned, and found him suffering intensely with pain in the abdomen. There was no appreciable tenderness on pressure, and no tympanites. Bowels had moved the day before from oil. Sixteen hours later he passed a pint of clotted blood.

Aug. 15. Slight tenderness of bowels, tongue moist and less flabby, temperature 102, pulse 106, pain in knee-joint entirely disappeared.

Aug. 16. Condition the same.

Aug. 17. Temperature and pulse both fallen.

Aug. 21. Temperature normal, and the case went on to recovery.

This case was one of a moist tongue throughout, no delirium, no tympanites, no tenderness of abdomen, except the day after the hemorrhage.

CASE V., male, aged 9 years. First examination Aug. 25, 1889. Had been not very well for two days, but seemed to have no fever till noon of this date. At 6, p. m., temperature

101 1-2, pulse 115, and rather weak, tongue with whitish coat, bowels moving regularly, complained of legs aching as though weary from walking.

Aug. 26, 8 a. m., temperature 100, pulse 105 and weak, tongue moist and furred; at 5 p. m. temperature 101, pulse 110, quite restless. Until September 3, there was gradual aggravation of these symptoms, and in addition some hebetude. On that day, his temperature at 6 p. m. was 103, pulse 127 and weak, skin dry, tongue dry, some delirium. His condition remained about the same for the next three days; on Sept. 7, the fever began to decrease, and on the 11, the temperature was normal. The fever lasted seventeen days, the tongue was never heavily coated, there was no abdominal tenderness, no diarrhœa, bowels normal every day throughout the case, some hebetude, and slight delirium for three or four days.

CASE VI., female, aged 19 years. Reported malaise for some days previous to my first visit, and probably there had been fever for four or five days. On July 28, 1889, temperature 101 1-2, pulse 98, tongue coated but moist, bowels had moved freely the day before, from a purgative, no abdominal tenderness, no diarrhœa, some muscular soreness.

July 29, 2 p. m., temperature 101, pulse 95, and regular, tongue moist and red at the edges, skin dry, headache, kidneys acting fairly well, had a very restless night.

July 30, 2:50 p. m., temperature 102, pulse 104, regular and quite strong, tongue moist, skin hot, headache severe, no tenderness of bowels, no tympanites, bowels moved in the morning from purgative taken the day previous.

July 31, 2 p. m., temperature 102, pulse 107, tongue moist, and all other symptoms as day before; muscular soreness disappearing.

Aug. 1, 3 p. m., temperature 103, pulse 112, tongue moist, lips parched and dry, skin hot.

Aug. 2, 9 a. m., temperature 102, pulse 110; at 2 p. m., temperature 102 1-2, pulse 114, some headache, but not severe.

Aug. 3, Condition almost identical with that of previous day.

Aug. 4, 10. a. m., temperature 102, pulse 114; 4 p. m., temperature 103, pulse 117, tongue moist, but covered with a thick pasty fur of brownish color, bowels constipated and very flat. From this date there was a progressive rise of temperature and acceleration of pulse with some mental hebetude until August 17th. On that day her temperature was 103 1-2 at 9 a. m., and at 5 p. m., 104 3-4.

Aug. 18. Condition the same.

Aug. 19. A slight decrease in temperature.

Aug. 20, 10 a. m., temperature 102 1-2; 5 p. m., 103 1-4.

Aug. 21, 10 a. m., temperature 102; 4 p. m., 103, considerable sweating in the morning. From this date the fever slowly decreased, and forty days from the seizure, the temperature was normal. In this case, the tongue was dry for four or five days; at no time was there tenderness of the bowels, tympany or rose-colored rash of typhoid. For a few days during the height of the fever, there was some hebetude and a slight degree of subsultus tendinum.

CASE VII, female, aged 20. First seen July 30, 1889. She had then been ailing for some three or four days, complaining of a tired feeling each evening, with restless nights, considerable headache and constipation. Had taken pills two days previously which produced copious action, but no movement since. Two hours before my visit she had a slight chill. At 5 p. m., temperature 101 1-2, pulse 96, tongue considerably coated, large and flabby; skin hot and dry. This case throughout its course, was in some respects, very like *case VI;* its duration was less, however, the temperature being normal on the twenty-second day, there was less weakness, and for several days slight diarrhoea and some tenderness of the bowels, no hebetude nor subsultus tendinum.

CASE VIII--Male, aged 26 years. On November 8th, the date of my first visit, his temperature was 101 1-2; at 4 p. m., pulse 84, tongue furred. Until the tenth day the temperature gradually increased, reaching then 104 3-4 in the evening. For four days it remained about the same and then descended quite abruptly, he being clear of fever the fifteenth day. At no time was there diarrhoea, tenderness of abdomen or tympanites.

CASE IX, male, aged 22 years, a brother of *case VI,* occupying the same house with her during and after her sickness. He was taken sick October 30, 1889. I shall not describe this case in detail. There was intense headache at the beginning; tenderness of the abdomen showed early; then decided tympanites; epistaxis on the sixth day; delirium of a low muttering form began on the fourteenth day; the tongue became dry and cracked, tympanites increased, pulse grew more rapid, temperature higher, delirium worse, until he sank into a state of coma; diarrhoea with the characteristic "pea soup" stools, finally incontinence of fæces, and death on the twenty-third day from perforation of bowel. There was none of the rose-colored rash in this case. You recognize in this description a malignant form of Typhoid fever.

CASE X was related to me by my lamented friend, Dr. E. N. Bailey. The patient was aged 26 years, a sister of *cases VI* and *IX*, and living not over fifty yards from *cases VI, VIII* and *IX*. She nursed *case VI* during the latter days of her sickness.

November 7, 1889, Dr. Bailey was first called. He found her with a temperature of 101, pulse 98. At the end of two weeks her evening temperature was 103, pulse rather weak, no tympany, no diarrhoea, and but little tenderness of bowels, no typhoid rash. On the seventeeth day of her illness she had hemorrhage from the bowels. This continued at intervals till it caused her death on the nineteenth day of her sickness.

CASE XI, male, aged 18 years, a brother of *cases VI, IX and X*. He was taken sick November 15, 1889. The disease ran the same course as *case IX*, with the same result. The Typhoid rose-colored rash was in this case very marked.

REMARKS: Cases I, II, V, VI, and VIII, are such as I have been accustomed to diagnose as continued fever, and conform to the generally accepted description of that disease throughout the South. I have abandoned any belief in its malarial origin, for in our vicinity quinine is not only fruitless of good, but is often fruitful of harm, and on the Cumberland Plateau we are probably as free from malaria as any part of the Nation. In the five cases mentioned, there were *none* of the characteristic symptoms of typhoid fever, neither epistaxis, nor tenderness of abdomen, nor tympanites, nor diarrhoea, nor rose-colored rash.

Of the other cases recited, *case III* occurring simultaneously and in close neighborhood to *case II*, exhibits one typhoid symptom—namely, tenderness of the bowels; *case IV* of the same household as *case II*, would form a typical case of continued fever, except for the one symptom—hemorrhage—unmistakably stamping it as typhoid. *Case VII*, would be classed as a typical and very mild case of continued fever, except for two symptoms—diarrhoea and tenderness of the bowels. *Case X*, in the immediate locality with *cases VI* and *VIII*, but occurring in the fall, has of the characteristic typhoid symptoms, tenderness of the abdomen, and exhausting hemorrhage from the bowels, finally ending in death. *Cases IX* and *XI*, of the same household as *case VI*, and in the immediate locality of *cases VIII* and *X*, occurred in the fall, and were distinguished from their first beginning as typical cases of malignant typhoid fever—cases whose type I am thankful to say is rarely seen in our town. But leaving out of view these two cases, the recital of fever cases that I have given to-day, forms a picture that I can duplicate from records of cases, selected hap-hazard, constantly occurring in my practice. The grouping will show the same likenesses and differences, the acceptably typical continued fever cases will preponderate, but elbow to elbow with them, occurring in the same household, in immediate vicinity, at the same time, will be other cases with one or more of the accepted markings of typhoid fever—and if I follow from midsummer to autumn, as in this recital, the typhoid symptoms will grow more pronounced with the season of leaf fall.

I have had no opportunity to verify by post mortem examinations my belief that enteric lesions occur in these non-typical, unclassified forms of fever, but Drs. J. P. Wall, of Tampa, Florida, George Dock, of Texas, and J. H. Vaneman, of Missouri, have reported the characteristic typhoid lesions at the autopsies of such, on cases resulting fatally. Nor need we be surprised that these *atypical* fever cases are shown to be enteric. Even if we grant that typhoid fever is produced by one specific germ, we know that in other diseases caused by a given micro-organism, protean manifestations are exhibited, ranging from the barely recognizable and mild to the undoubted and fatal aspects. But if, as seems more probable to me, enteric fever is due to a mixed infection, as Vaughn, of Michigan, suggests,* then we should naturally expect the great variety of symptoms, the difference in types, and the wide range of severity which we find.

---

*See "Some New Bacteral Poisons" by Victor C. Vaughn, of Ann Arbor, Michigan.

